# Safety of healthy subjects in first-in-human multiple-dose studies: A pooled analysis 

**DOI:** 10.5414/CP203760

**Published:** 2020-09-29

**Authors:** David Jung, Inka Valeska Braun, Georg Wensing

**Affiliations:** 1 Experimental Medicine Cardiovascular and; 2 Clinical Pharmacology Cardiovascular, Bayer AG, Wuppertal, Germany

**Keywords:** healthy subjects, first-in-human, safety, multiple dose

## Abstract

Purpose: Reflecting the extended scope of the valid EMA regulation, this analysis intends to contribute to the knowledge about risk for participants in first-in-human (FiH) multiple-dose studies. Materials and methods: All FiH multiple-dose studies in healthy subjects performed by the Bayer Department of Clinical Pharmacology, Cardiovascular, between 2006 and 2019 were analyzed. Study reports were reviewed for study designs, demographics, treatment-emergent adverse events (TEAEs), and safety laboratory results above the 1.5-fold of the upper limit of normal (aspartate aminotransferase (AST), alanine aminotransferase (ALT), creatine kinase (CK), amylase, lipase, glutamate dehydrogenase (GLDH), gamma glutamyl transpeptidase (GGT), total bilirubin, and creatinine in serum), and data were analyzed. Results: 12 out of 16 studies were included. Indications for development were cardiovascular (7), pulmonary (3), kidney (1), and hematological (1) diseases. 496 healthy male subjects (mean age 33.8 years, mean BMI of 24.7 kg/m^2^) received treatment (370 active, 126 placebo). 293 subjects had at least 1 TEAE (59.1%): 231 (62.4%) after active treatment and 126 (49.2%) after placebo. Subjects with a maximum TEAE intensity of moderate did not differ between active and placebo. The only severe TEAE was unrelated to the study, the only serious TEAE on active treatment was not considered drug-related. Subjects had a significantly higher relative risk on active treatment versus placebo to experience an overall TEAE. No relevant differences between active and placebo for the analyzed laboratory increases were seen. Conclusion: Subjects were not exposed to an undue risk in the analyzed studies. Adverse events and laboratory value increases occur frequently under placebo treatment. The results can help in the risk stratification for and interpretation of other phase I studies.

**What is known about this subject**

- Safety of healthy subjects in first-in-human multiple-dose studies has become subject to recent regulation and current discussion.
- Published data about safety in first-in-human multiple-dose studies is lacking.

**What this study adds**

- Provides pooled safety data of first-in-human multiple-dose trials in healthy subjects.
- Supports thereby risk evaluation of first-in-human multiple-dose trials with healthy subjects by broadening the published database.

## Introduction 

An important extension in the scope of the valid guideline for early clinical trials with investigational medicinal products of the European Medicines Agency (EMA) is the coverage of studies beyond single-dose first-in-human (FiH) trials [1]. While previous regulation focused on determination of a safe starting dose and dosing selection in single-dose FiH trials [2], considerations for the ensuring of safety during the first multiple-dose administration in humans have become an important part of the new regulation. The death case in the Bial trial in 2016, which happened in the multiple-dose part of this FiH study [3], illustrates dramatically the importance of paying particular attention to safety in this step of early clinical development. Potential accumulation might become evident and relevant for target-related side effects and can possibly also lead to off-target effects [4]. Despite the general perception of an acceptable risk in early clinical trials in healthy subjects, systematic data for risk determination remain rare [5, 6] especially in regard to the different types of early clinical studies. 

In order to contribute to the general knowledge for risk stratification, we analyzed safety data of our in-house FiH multiple-dose studies in healthy subjects for all compounds that went into human development between 2006 and 2019. Systematic safety data from preceding FiH single-dose studies has recently been published, and an overall low risk for participants was shown [7]. A similar result was also demonstrated for previous FiH studies [8]. The aim of this analysis was to determine if a relevant increase of risk occurs at the important step from first single- to multiple-dose administration in humans. 

## Materials and methods 

All FiH multiple-dose studies in healthy subjects performed by the Department of Clinical Pharmacology, Cardiovascular, Bayer AG Wuppertal, between January 2006 and May 2019 were analyzed for inclusion. All studies were approved by the responsible independent and competent Ethics Committee of the Medical Council North Rhine (Düsseldorf) and the German competent authority. Informed consent was obtained from all individual subjects included in the studies, and all subjects were obliged as per protocol not to take part in different clinical studies without a minimum pause of 3 months in between. Formulations used for the active drug and placebo were identical in appearance (size, shape, color). 

Study reports were reviewed to compile the data used in this analysis. Study designs, demographics, treatment-emergent adverse events (TEAEs), and safety laboratory results for AST (aspartate aminotransferase), ALT (alanine aminotransferase), CK (creatine kinase), amylase, lipase, GLDH (glutamate dehydrogenase), GGT (gamma glutamyl transpeptidase), total bilirubin, and creatinine in serum were systematically analyzed. Numbers, percentages per group, and relative risk of occurrence were calculated. TEAEs were sorted by the Medical Dictionary for Regulatory Activities (MeDRA, current version, respectively) primary system organ class and preferred term, intensity was categorized into mild, moderate and severe, and into serious and non-serious. Laboratory results were analyzed regarding the occurrence of values above 1.5-fold of the upper limit of normal during the in-house phase of the studies. 

## Results 

### Overall studies 

Of all 16 analyzed studies, 12 studies had reported data at the timepoint of analysis and were included, 4 studies were still ongoing and were not included. All included studies tested small molecules and only multiple-dose escalation, parallel group comparison, single blind, placebo-controlled designs were used. Three of the included studies had comparator arms and/or interaction parts that were all excluded from this analysis. Routes of administration were in 11 cases oral (immediate-release tablets), and in 1 case intranasal (solution). The number of dose steps was 3 or 4 in most of the studies, only 1 study had 5 dose steps. Escalation factors between daily doses ranged from 1 (once versus twice daily dosing of the same daily dose) to 2.5, the ratios between the highest and lowest daily doses ranged from 3 to 10. Indications for development were for 7 compounds cardiovascular, for 3 pulmonary, for 1 kidney, and for 1 hematology diseases. In all studies, the investigated doses were justified by the estimated dose range for later therapeutic use, mostly starting at the lower and escalating up to the upper boundary as evidenced by results of a FiH study and/or preclinical studies [9]. None of the studies was terminated prematurely. In 1 study, administration in the planned highest dose step was assessed as not well tolerated (vasoactive compound with exaggerated effects and/or response related to the mode of action of vasodilation). In the others, good tolerance was seen up to the highest dose step. At cut-off, 6 compounds were still under development (2 in phase III, 4 in phase II). The other 6 were terminated, thereof 5 in phase II. In 1 case, the results of the included multiple-dose escalation study led to termination thereafter (insufficient dose-response relationship). 

### Included subjects and treatments 

All studies only enrolled overtly healthy male subjects without clinically relevant deviations of laboratory values, including renal and hepatic function parameters and hematology and of vital signs and ECG. Use of concomitant medication was not allowed except for single doses of ibuprofen and/or paracetamol. The included subjects were all Caucasians with a mean age of 33.8 years and a mean body mass index (BMI) of 24.7 kg/m^2^. 496 subjects received treatment (370 active and 126 placebo treatments). The relation between active and placebo was similar (~ 3 : 1) in all studies. For details of treated subjects in different indications see Table 1. 

Numbers of consecutive treatment days ranged between 5 and 21, and 5 studies had a preceding single-dose treatment followed by a non-treatment period before starting multiple-dose treatment. 12 subjects (8 active, 4 placebo) dropped out after treatment initiation before the planned end of the treatment (for details of drop-outs see below). 

### Adverse events 

Overall, 621 TEAEs occurred during the studies and were assigned to 172 adverse event (AE) terms. The most frequent TEAE was headache (14.8% of all TEAEs, for further TEAEs see Table 2). 499 TEAEs were reported in treatment with active drug and 122 in placebo. For 293 subjects, at least 1 TEAE was reported (59.1%), thereof in 231 (62.4%) subjects after active treatment and in 62 (49.2%) after placebo treatment. Most of these subjects had a mild intensity (54.2% of all subjects), 4.6% of all subjects a moderate, and only 1 subject a severe maximum intensity of a TEAE. Percentages of subjects with a maximum TEAE intensity of moderate were comparable between active and placebo treatment. The rate of subjects with a TEAE in active treatment was higher at higher dose steps with a clear difference to placebo from dose step 2 and higher. However, the proportion and the overall percentage of subjects within a dose group with a maximum TEAE intensity of moderate were not higher at higher dose steps and did not differ between active and placebo treatment. For details of distribution of dose steps see Table 3. 

**Figure 1.**
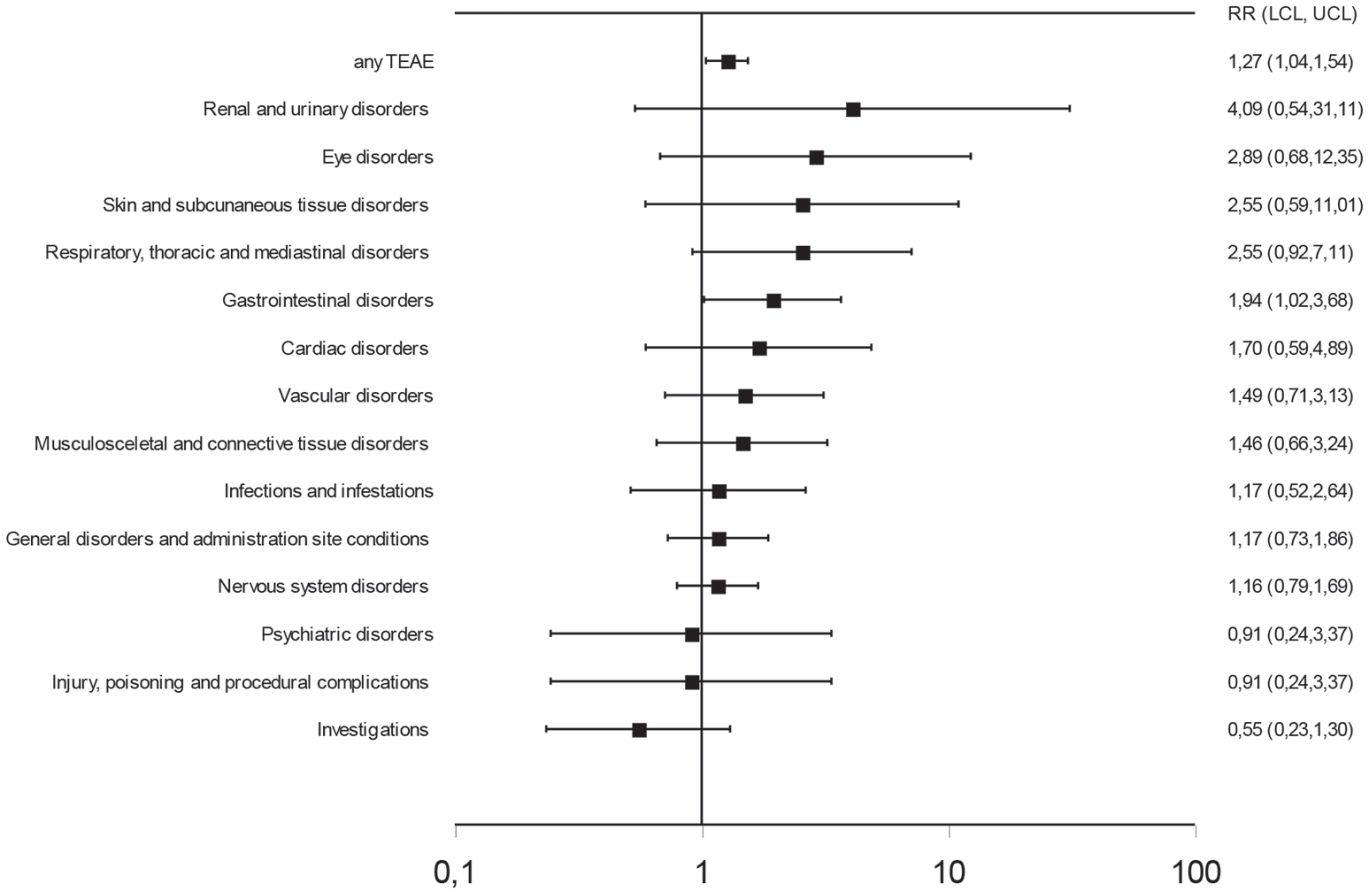
Relative risks (RR) with lower (LCL) and upper (UCL) limits of the 95% confidence interval for treatment-emergent adverse events (TEAEs) of system organ class affected in > 2% of all treated subjects.

The most affected system organ class was the central nervous system, without showing a clear difference between active treatment and placebo. The system organ class that showed the highest percentage of affected subjects with a noticeable difference between active treatment and placebo was the gastrointestinal system. For details of affected system organ classes see Table 4. A significantly higher relative risk for subjects on active treatment versus placebo for experiencing a TEAE was shown for an overall TEAE and for a TEAE of the gastrointestinal system. For details see Figure 1. 

**Severe and serious adverse events and treatment discontinuations**

The only reported severe TEAE was a planned minor surgical intervention after the last visit in a subject who had received placebo. This TEAE was therefore unrelated to the study. 

A total of 3 serious adverse events (SAEs) were reported during the studies, none of which was considered drug related: 1 syncope (moderate) in dose step 1 of 3 in a subject on active treatment with a non-vasoactive compound with a belatedly revealed medical history finding of repetitive syncope, 1 ALT increase (moderate) 37 days after last dosing with placebo due to alcohol consumption, and 1 previously planned minor surgical intervention (severe) after placebo treatment. The reasons for seriousness were medical importance concerning the ALT increase and syncope and hospitalization for the planned surgery. Two SAEs resolved completely, the ALT increase was improving at the last contact to the investigator (subject was lost to follow-up). 

Six discontinuations of treatment due to TEAEs were reported. Two subjects discontinued due to moderate TEAEs on active drug: 1 sinus tachycardia 8 days after first dosing (considered drug-related), 1 syncope that was also reported as SAE (for details see above). Four subjects had mild TEAEs, 3 of which were on active drug and 1 on placebo: 1 swelling of right cheek due to tooth inflammation (not considered drug-related), 1 redness of both eyes on placebo (not drug-related), 1 exanthema of buttock and extremities on active drug (considered drug-related), and 1 trichomoniasis (not drug-related). Each of these TEAEs resolved completely. Of the further 6 subjects who discontinued, 5 withdrew consent, and 1 had a belated medical history finding that would have precluded his inclusion. 

### Laboratory 

The results showed no relevant differences between active treatment and placebo in the percentages of affected subjects by increases of amylase, lipase, CK, ALT, AST, GLDH, bilirubin, AP, GGT and creatinine above 1.5-fold of the upper limit of normal during the in-house phases of the studies. Relative risks for such increase were higher under active treatment as compared to placebo for lipase, ALT, AST, GLDH, and GGT without reaching significance level for any of them and showed high variability. For details of subjects with laboratory value increases above 1.5-fold see Table 5. 

## Discussion 

Overall, the results show that the participating subjects in our studies were not exposed to an undue risk for harm. A low number of severe and serious TEAEs occurred, and the only serious TEAE on active drug was not considered drug-related, and the subject fully recovered. 

The likelihood of experiencing an AE was higher under active treatment compared to placebo and was higher at higher doses. This, as well as the nature of the AEs (headache, flushing, nasal congestion, ocular hyperemia are among the most frequent TEAEs), reflects and can be well explained by the profile of the included predominantly vasoactive compounds: They mostly caused mode of action-related adverse effects closely related to the exposure and could be well monitored. 

Regarding the low overall risk, our results are in line with published metadata about phase I trials with healthy subjects [6, 10], whereas the differences of included compounds and studies have to be considered. 

An aim of this study was to compare these multiple-dose studies to the published safety data of the single-dose FiH studies of a similar time period [7], which included 10 compounds tested in the presented multiple-dose studies; no generally higher risk for harm could be detected for multiple-dose in comparison to single-dose treatment, and the nature of the TEAEs was comparable, as expected. The results were similar regarding the differences between active drug and placebo in the percentage of affected subjects by TEAEs (after single dose: 42.1% on active, 34% on placebo). The higher total percentages in the multiple-dose studies in active and placebo groups can be explained by the longer treatment periods, which by nature go along with a higher likelihood of experiencing an event. 

However, in the multiple-dose studies, the tendency for a higher rate of subjects affected by TEAEs in higher dose steps on active treatment was more pronounced than in the single-dose studies. This can be interpreted as an indicator that substance-related adverse effects were especially revealed under higher multiple doses. 

The interpretation of the comparison between placebo-controlled single- and multiple-dose studies is in accordance with previous publications that also show higher rates for TEAEs after multiple doses in active and placebo groups and also conclude that especially multiple-dose treatment can reveal compound-specific AE profiles [11]. 

Regarding the results for the laboratory changes, no relevant differences are shown between active and placebo treatment. Noticeably, a remarkable number of subjects had increases of the analyzed parameters during the in-house phase under placebo. This indicates that factors beyond treatment were causative for laboratory changes during the studies. Various aspects have been discussed in the literature especially in regard to liver enzyme increases in this context and are mostly suggested to be related to hospitalization effects but also to demographics, individual predisposition, and physiological regulation [12, 13, 14]. Our data are in line with other recently published data [15] and support the assumption that factors like the ones mentioned, not associated to the study drugs, in fact play a role in causing laboratory changes during clinical studies. 

In our studies, only subjects were included without clinically relevant laboratory deviations at the time of inclusion. On this background, the observed frequent increases in the placebo group during treatment further support the commonly accepted recommendation to follow strict eligibility rules for such studies [16]. This improves not only subject safety but could thus also prevent the inclusion of subjects with predisposition to show further laboratory elevations during treatment and thereby impair the assessment of the placebo group as a reasonable control to detect signals of organ toxicity. 

With regard to AEs in the placebo group, our data, again similar to other analyses [15], show that also AEs are reported to a remarkable amount during placebo treatment. This data can help to improve the understanding of what we can expect in placebo groups and thereby support the interpretation of the safety results of healthy subject studies. 

This analysis has some limitations. Main limitations are the number of included subjects, which is not high enough to reveal unlikely side effects, and the comparatively homogenous combination of mostly vasoactive compounds, which by nature restricts general applicability. 

## Conclusion 

Subjects were not exposed to an undue risk in the analyzed multiple-dose FiH studies. The risk for harm was similarly low as in our single-dose FiH studies, and therefore the assumption that a higher risk for subjects after multiple dosing due to effects of accumulation cannot be supported by our data. AEs and changes in laboratory values occur to a relevant amount under placebo treatment, which should be considered and can help in the interpretation of other study results. 

## Funding 

All trials included in the analysis were funded by Bayer AG. 

## Conflict of interest 

All authors are employees of Bayer AG. 

**Table 1.** Treatments sorted by indications.

Indication	Subjects	Subjects receiving active drug	Subjects receiving placebo
Cardiovascular	296	220	76
Pulmonary	113	85	28
Hematology	48	36	12
Kidney	39	29	10
**Overal**l	**496**	**370**	**126**

**Table 2.** TEAEs with > 1% proportion of all TEAEs.

AE term	% of all TEAEs (100% = 621)
Headache	14.8
Dyspepsia	4.5
Flushing	4.3
Fatigue	2.7
Nasopharyngitis	2.3
Nasal congestion	2.3
Ocular hyperemia	2.1
Catheter-site pain	2.1
Back pain	2.1
Nausea	1.6
Vessel puncture-site reaction	1.6
Micturition urgency	1.6
Palpitations	1.4
Diarrhea	1.4
Catheter-site swelling	1.4
Thirst	1.4
Sinus tachycardia	1.3
Feeling hot	1.3
Dizziness	1.3
Restlessness	1.3
Myalgia	1.1
Application-site erythema	1.0
Oral herpes	1.0
Subcutaneous hematoma	1.0
Paranesthesia	1.0

TEAE = treatment-emergent adverse event; AE = adverse event.

**Table 3.** Distribution of subjects with at least 1 TEAE to dose steps.

Dose group	Subjects treated	Overall	TEAE maximum intensity		
			Mild	Moderate	Severe
		Subj. with TEAE % in group	Affected subj. % of affected % of treated	Affected subj. % of affected % of treated	Affected subj. % of affected % of treated
Total	496	293 59.1%	269 91.8% 54.2%	23 7.8% 4.6%	1 0.3% 0.2%
Active drug	370	231 62.4%	213 92.2% 57.6%	18 7.8% 4.9%	0
Placebo	126	62 49.2%	56 90.3% 44.4%	5 8.1% 4.0%	1 1.6% 0.8%
Dose step 1	101	54 53.5%	48 88.9% 47.5%	6 11.1% 5.9%	0
Dose step 2	101	65 64.4%	63 96.9% 62.4%	2 3.1% 2.0%	0
Dose step 3	100	65 65.0%	56 86.2% 56.0%	9 13.8% 9.0%	0
Dose step 4	59	44 74.6%	43 97.7% 72.9%	1 2.3% 1.7%	0
Dose step 5	9	3 33.3%	3 100% 33.3%	0	0

TEAE = treatment-emergent adverse events.

**Table 4.** Distribution of subjects with at least 1 TEAE to primary system organ classes.

Number of subjects with at least 1 TEAE	Total (n = 496)	Active (n = 370)	Placebo (n = 126)
Overall	293 (59.1%)	231 (62.4%)	62 (49.2%)
Nervous system disorders	119 (24.0%)	92 (24.9%)	27 (21.4%)
General disorders and administration site conditions	84 (16.9%)	65 (17.6%)	19 (15.1%)
Gastrointestinal disorders	67 (13.5%)	57 (15.4%)	10 (7.9%)
Vascular disorders	43 (8.7%)	35 (9.5%)	8 (6.3%)
Musculoskeletal and connective tissue disorders	37 (7.5%)	30 (8.1%)	7 (5.6%)
Respiratory, thoracic, and mediastinal disorders	34 (6.9%)	30 (8.1%)	4 (3.2%)
Infections and infestations	31 (6.3%)	24 (6.5%)	7 (5.6%)
Cardiac disorders	24 (4.8%)	20 (5.4%)	4 (3.2%)
Investigations	21 (4.2%)	13 (3.5%)	8 (6.3%)
Eye disorders	19 (3.8%)	17 (4.6%)	2 (1.6%)
Skin and subcutaneous tissue disorders	17 (3.4%)	15 (4.1%)	2 (1.6%)
Renal and urinary disorders	13 (2.6%)	12 (3.2%)	1 (0.8%)
Injury, poisoning, and procedural complications	11 (2.2%)	8 (2.2%)	3 (2.4%)
Psychiatric disorders	11 (2.2%)	8 (2.2%)	3 (2.4%)
Ear and labyrinth disorders	7 (1.4%)	5 (1.4%)	2 (1.6%)
Reproductive system and breast disorders	3 (0.6%)	3 (0.8%)	0
Metabolism and nutrition Disorder	2 (0.4%)	0	2 (1.6%)
Blood and lymphatic system disorders	1 (0.2%)	0	1 (0.8%)

TEAE = treatment-emergent adverse events.

**Table 5.** Subjects with values ≥ 1.5 ULN during in-house phase and RR with LCL and UCL limit of 95% confidence interval.

	Total (n = 496)	Active (n = 370)	Placebo (n = 126)	RR [LCL – UCL] (> 1: placebo better < 1: active better)
Amylase	6 (1.2%)	4 (1.1%)	2 (1.6%)	0.7 [0.1 – 3.7]
Lipase	33 (6.7%)	28 (7.6%)	5 (4.0%)	1.9 [0.8 – 4.8]
Creatine kinase	25 (5.0%)	16 (4.3%)	9 (7.1%)	0.6 [0.3 – 1.3]
ALT	23 (4.6%)	18 (4.9%)	5 (4.0%)	1.2 [0.5 – 3.2]
AST	5 (1.0%)	5 (1.4%)	0	3.4 [0.2 – 61.9]
GLDH	33 (6.7%)	28 (7.6%)	5 (4.0%)	1.9 [0.8 – 4.8]
Bilirubin	3 (0.6%)	2 (0.5%)	1 (0.8%)	0.7 [0.1 – 7.4]
AP	0	0	0	
GGT	2 (0.4%)	2 (0.5%)	0	1.4 [0.1 – 30.0]
Creatinine	0	0	0	

ULN = upper limit of normal; RR = relative risk; LCL = lower limit of 95% confidence interval; UCL = upper limit of 95% confidence interval.

## References

[1] CHMP (Committee for Medicinal Products for Human Use). Guideline on strategies to identify and mitigate risks for first-in-human and early clinical trials with investigational medicinal products. 20 July 2017; EMEA/CHMP/SWP/28367/07 Rev.1.

[2] CHMP (Committee for Medicinal Products for Human Use). Guideline on Requirements for first-in-man clinical trials for potential high-risk medicinal products. London, March 22, 2007; EMEA/CHMP/SWP/28367/2007..

[3] Kerbrat A Ferré JC Fillatre P Ronzière T Vannier S Carsin-Nicol B Lavoué S Vérin M Gauvrit JY Le Tulzo Y Edan G Acute neurologic disorder from an inhibitor of fatty acid amide hydrolase. N Engl J Med. 2016; 375: 1717–1725. 27806235 10.1056/NEJMoa1604221

[4] Chaikin P The Bial 10-2474 Phase 1 Study – A drug development perspective and recommendations for future first-in-human trials. J Clin Pharmacol. 2017; 57: 690–703. 28387940 10.1002/jcph.889

[5] Karakunnel JJ Bui N Palaniappan L Schmidt KT Mahaffey KW Morrison B Figg WD Kummar S Reviewing the role of healthy volunteer studies in drug development. J Transl Med. 2018; 16: 336. 30509294 10.1186/s12967-018-1710-5 PMC6278009

[6] Johnson RA Rid A Emanuel E Wendler D Risks of phase I research with healthy participants: A systematic review. Clin Trials. 2016; 13: 149–160. 26350571 10.1177/1740774515602868 PMC4783291

[7] Jung D Boettcher MF Wensing G How safe are our studies? Analysis of adverse events in Bayer First-in-Human trials from 2006 to 2016. Int J Clin Pharmacol Ther. 2020; 58: 10–20. 31746730 10.5414/CP203390

[8] Wensing G Ighrayeb IA Boix O Böttcher M The safety of healthy volunteers in First-in-Man trials – an analysis of studies conducted at the Bayer in-house ward from 2000 to 2005. Int J Clin Pharmacol Ther. 2010; 48: 563–570. 20860909 10.5414/cpp48563

[9] Jung D Mueck W Wensing G First in Human (FiH) studies in healthy volunteers – a 10 years review. Front. Pharmacol. Conference Abstract: EUFEMED; 2017.

[10] Emanuel EJ Bedarida G Macci K Gabler NB Rid A Wendler D Quantifying the risks of non-oncology phase I research in healthy volunteers: meta-analysis of phase I studies. BMJ. 2015; 350: h3271. 26115663 10.1136/bmj.h3271 PMC4482145

[11] Lutfullin A Kuhlmann J Wensing G Adverse events in volunteers participating in phase I clinical trials: a single-center five-year survey in 1,559 subjects. Int J Clin Pharmacol Ther. 2005; 43: 217–226. 15906587 10.5414/cpp43217

[12] Cai Z Christianson AM Ståhle L Keisu M Reexamining transaminase elevation in Phase I clinical trials: the importance of baseline and change from baseline. Eur J Clin Pharmacol. 2009; 65: 1025–1035. 19554320 10.1007/s00228-009-0684-x

[13] Rosenzweig P Miget N Brohier S Transaminase elevation on placebo during phase I trials: prevalence and significance. Br J Clin Pharmacol. 1999; 48: 19–23. 10383555 10.1046/j.1365-2125.1999.00952.x PMC2014876

[14] Merz M Seiberling M Höxter G Hölting M Wortha HP Elevation of liver enzymes in multiple dose trials during placebo treatment: are they predictable? J Clin Pharmacol. 1997; 37: 791–798. 9549632 10.1002/j.1552-4604.1997.tb05626.x

[15] Young TC Srinivasan S Vetter ML Sethuraman V Bhagwagar Z Zwirtes R Narasimhan P Chuang T Smyth BJ A systematic review and pooled analysis of select safety parameters among normal healthy volunteers taking placebo in phase 1 clinical trials. J Clin Pharmacol. 2017; 57: 1079–1087. 28510323 10.1002/jcph.913 PMC5573961

[16] Breithaupt-Groegler K Coch C Coenen M Donath F Erb-Zohar K Francke K Goehler K Iovino M Kammerer KP Mikus G Rengelshausen J Sourgens H Schinzel R Sudhop T Wensing G Who is a ‘healthy subject’? Consensus results on pivotal eligibility criteria for clinical trials. Eur J Clin Pharmacol. 2017; 73: 409–416. 28064353 10.1007/s00228-016-2189-8 PMC5350217

